# Comparative evaluation of sperm parameters in Italian (*Apis mellifera ligustica*) and Africanized (*Apis mellifera*) honeybee drones from the Caatinga biome

**DOI:** 10.1590/1984-3143-AR2025-0113

**Published:** 2026-04-03

**Authors:** Lilian Leal Dantas, Andréia Maria da Silva, Leandro Alves da Silva, Pedro Augusto Pinheiro Brito, Yuri Gonçalves Matos, Romário Parente Santos, Kátia Peres Gramacho, Alexandre Rodrigues Silva

**Affiliations:** 1 Laboratório de Conservação de Germoplasma Animal, Centro de Ciências Agrárias, Universidade Federal Rural do Semi Árido, Mossoró, RN, Brasil; 2 Núcleo de Capacitação Tecnológica em Apicultura, Universidade Federal Rural do Semi Árido, Mossoró, RN, Brasil

**Keywords:** beekeeping, biobank, conservation, polinators

## Abstract

Bees are essential pollinators with species differing morphologically and physiologically. Understanding the variations in reproductive parameters between phenotypes is crucial. This study compares the sperm characteristics of Italian (*Apis mellifera ligustica*) and Africanized drones (*Apis mellifera L.*) raised in the Caatinga biome. Nine sexually mature Italian drones and sixteen Africanized drones from different colonies were used. Semen was collected using the endophallus eversion technique and diluted in saline solution (1:20). The parameters analyzed included: motility (optical microscopy), sperm viability (Hoechst 33342; propidium iodide), functional integrity of the plasma membrane (hypo-osmotic test), morphology and morphometry (Rose Bengal), and scanning electron microscopy. The results were expressed as mean ± standard error. Statistical analyses included the Shapiro-Wilk test to the normality of residuals and the Bartlett test to verify homoscedasticity. Comparisons between groups were performed using the Mann-Whitney and Student's t-tests (P < 0.05). Both phenotypes presented 90% sperm motility with viability of 82.4 ± 2.5% for Italians and 81.1 ± 2.4% for Africanized ones; the functional integrity of the plasma membrane was 93.4 ± 1.8% and 91.6 ± 1.5%, respectively. Regarding morphology, the percentage of normal sperm was 10.89 ± 1.66% for Italian and 12.06 ± 1.01% for Africanized, with the curled tail being the most predominant feature of sperm morphology. No statistically significant differences (P > 0.05) were observed for the above-mentioned parameters. Sperm head morphometry was significantly larger (P < 0.05) in Italian (10.04 ± 0.03 µm) compared to Africanized (9.33 ± 0.04 µm). Scanning electron microscopy analysis revealed no ultrastructural differences between phenotypes. In conclusion, there is a high degree of similarity in sperm parameters of both phenotypes under the same environmental conditions, indicating the feasibility of applying similar reproductive strategies.

## Introduction

Pollination is essential for maintaining ecosystem balance. Bees and other insects are responsible for approximately 75% of the fruit and seed crops consumed by humans, making them fundamental for biodiversity preservation (FAO, 2018). However, in recent decades, a decline in these pollinators has been observed due to the loss, alteration, and fragmentation of natural habitats (Kline and Joshi, 2020), the intensification of agricultural practices and monoculture (Hemberger et al., 2021), exposure to parasites and pesticides (Bird et al., 2021), rising climate temperatures (Soroye et al., 2020), and the introduction of invasive species (Page and Williams, 2023). In this context, it is essential to understand the biology and reproductive parameters of different bee species to promote management adjustments and improve germplasm conservation protocols, expanding knowledge about the reproduction of these species.

A limiting factor for bee reproduction is sperm quality (Yániz et al., 2020, Zhao et al., 2021), as the queen will store viable sperm in her spermatheca for several years (Degueldre and Aron, 2023). This will determine the queen's reproductive success and the colony's productivity (Pettis et al., 2016), as well as the success of instrumental insemination (Khan et al., 2022; Güler et al., 2022). In this context, the male's fertility potential can influence the overall health of the colony (Murray et al., 2023).

Studies indicate that there may be differences in semen quality between males from the same species but with different phenotypes, such as birds (Ayeneshet et al., 2024), pigs (Wysokińska and Szablicka, 2021) and other mammals. For bees, comparisons on the reproductive quality of Caucasian (*Apis mellifera caucasica*) and Italian honeybees (*Apis mellifera ligustica*) conducted in Ankara, Turkey, revealed that the Caucasian drones present significantly greater body weight, ejaculate volume, number of sperm in the seminal vesicles, in addition to a greater ejaculation proportion, ejaculation efficiency and semen collection efficiency than the Italian phenotype (Kahya and Gençer, 2023). These findings indicate that there may be differences in reproductive parameters between the other honeybee phenotypes. However, comparative studies are scarce. Main studies are focused on characterizing sperm details of specific individuals as reported for Africanized drones (*Apis mellifera L.*) in the Caatinga biome in Brazil (Morais et al., 2022) and for drones of Italian honeybees (*Apis mellifera ligustica*) in the Campania region of Italy (Power et al., 2019).

Despite the genetic influence, environmental factors may also contribute to differences in reproductive and sperm characteristics, even among drones with similar genetic origins and geographical distribution (Taha and Alqarni, 2013; Kahya and Gençer, 2023). Thus, the hypothesis guiding this research is that drones from different phenotypes, raised under similar environmental conditions in the Caatinga biome, may present significant variations in sperm quality, considering that genetic, adaptive, and physiological factors directly influence the production, structure, and functionality of sperm cells (Rangel and Fisher, 2019). Given the practical importance of this topic, identifying possible differences in sperm parameters between phenotypes would have important applications for the reproductive management of colonies in the Caatinga biome. It would allow the selection of phenotypes with reproductive advantages and greater suitability for genetic improvement programs, instrumental insemination, or germplasm conservation. On the other hand, the absence of significant differences would also be relevant, as it would indicate that both phenotypes are equally viable from a reproductive perspective, providing greater flexibility in management practices.

Therefore, the objective of this study is to compare the semen quality of Italian honeybees (*Apis mellifera ligustica*) with that of Africanized drones (*Apis mellifera L.*) raised in the Caatinga biome, in terms of sperm motility, morphology, morphometry, ultrastructure, and membrane integrity and functionality.

## Materials and methods

The study was conducted with honeybee colonies from the UFERSA Experimental Station (5º03‟37” S and 37º23‟50” W, located in Mossoró, Rio Grande do Norte, Brazil). The experiments were carried out at the Animal Germplasm Conservation Laboratory (LCGA) of the Federal Rural University of the Semi-Arid (UFERSA). The study was conducted from July/2024 during the rainy period of the Caatinga biome. As this is a study with insects, consequently from the phylum Arthropoda, bees do not fall under Brazilian Law No. 11,794, of October 8, 2008, which only covers animals from the phylum Cordatha, thus waiving the need for authorization by an ethics committee.

### Animals

Drones were captured in different colonies located in the same apiary at the UFERSA Experimental Station. A total of 25 honeybee drones were used in the experiment, 9 Italian drones (*Apis mellifera ligustica*) and 16 Africanized (*Apis mellifera L.*), approximately 25 days old. They were collected at the entrance of the hive using a bee escape in which they were retained. In these, the drones were evaluated for maturity, being considered mature when they presented complete eversion of the tip of the endophallus with the presence of semen. Only mature individuals were used for the experiment (Collins and Donoghue, 1999).

### Semen collection

The semen collection was performed using the standard collection technique of eversion of the endophallus (Cobey et al., 2013). Stimulation was performed manually by applying gentle pressure to the drone's abdomen, which induced partial exposure of the endophallus, followed by complete eversion, exposing the semen at the tip of the endophallus, close to a white mucous secretion (Mendes, 2008). Approximately 1 μL of semen was collected using a HARBO instrumental insemination syringe. Subsequently, the samples were diluted in saline solution at a ratio of 1:20 for analysis.

### Sperm motility analysis

The sperm motility analysis (%) was performed using a 5µL aliquot that was placed on a preheated glass slide (34ºC), observed under phase-contrast microscopy (10x). It was estimated as a percentage from 0 to 100% depending on the number of spermatozoa in motion (Collins, 2005).

### Sperm viability

Sperm viability (%) was performed using a fluorescent marker. Thus, a 5μL aliquot of semen was incubated at 34ºC for 10 minutes in a fluorescent solution composed of 1 μL of propidium iodide (PI; Sigma-Aldrich, Co., St Louis, MO, USA) and 5 μL of Hoechst 33342 (H-342; Sigma-Aldrich, St Louis, MO, USA). The samples were then evaluated using an epifluorescence microscopy (40x; Episcopic Fluorescent Attachment “EFA” Halogen Lamp Set; Leica, Kista, Sweden). A total of 100 sperm cells were counted for each sample. Sperm cells with heads marker in blue were classified as viable, with intact membranes, while those stained partially or completely in red were classified as non-viable, with non-intact membranes (Hopkins and Herr, 2010).

### Functional integrity of the sperm membrane

To assess the functional integrity of the sperm membrane, the hypo-osmotic swelling test (HOST) was used, based on the curling and swelling of the tails. For this, 5 μL of semen was incubated in 45 μL of a hypo-osmotic solution of 0 mOsm/kg (distilled water) for 60 minutes at 34 °C (Nur et al., 2012). After the incubation period, 10 μL of the hyposmotic solution containing the sperm were deposited on a glass slide, covered with a coverslip, and evaluated using specific phase-contrast microscopy (40x). A total of 100 sperm cells per slide were evaluated in randomly selected microscopic fields, and the percentage of sperm with coiled tails was calculated (Nur et al., 2004).

### Sperm morphology and morphometry

To evaluate sperm morphology, slides stained with Rosa Bengala (Cromato, SP, Brazil) were prepared using 5 µL of semen in 45 µL of stain, incubated for 2 hours at room temperature. Subsequently, a 10 µL aliquot was placed on a glass slide and covered with a cover glass. After slide preparation, 100 cells per drone were counted in randomly selected fields under light microscopy (40x). Spermatozoa were classified as normal and abnormal when defects were present in the tail or head (Tarliyah et al., 1999).

Sperm morphometry was performed using morphology slides stained with Rose Bengal. From each drone, seven to eleven spermatozoa were analyzed, totaling 300 images that were captured using a light microscope (40x) with the Leica LAS V.4.13 software (Leica Microsystems, Wetzlar, Germany). In each image, the sperm components were measured using an image analysis software (ImageJ software, Wayne Rasband, National Institute of Health, Maryland, USA). The morphometric parameters measured included sperm head, flagellum, and total length (Gontarz et al., 2016).

### Scanning electron microscopy (SEM)

The samples were processed for SEM following the protocol described by Santos et al. (2022) with some modifications. For evaluation of sperm on a three-dimensional surface, an adaptation was made to the protocol using samples fixed on a morphology slide with Rose Bengal for 24 h. The spermatozoa were dried in ar and the coverslips were fastened with an adhesive tape on brass stubs and then gold coated in the metallizer Sputter Coater Quorum, Q150R ES, Reino Unido and were observed under a scanning electron microscope (Tescan®, Type VEGA 3 LMU, Nº VG13671479, 50/60 Hz; Brno, Czech Republic). The samples were then processed for evaluation by SEM in the laboratory of the Center for Research in Plant Sciences of the Semiarid, UFERSA. In each image, the sperm components were measured using image analysis software (ImageJ, Wayne Rasband, National Institutes of Health, Maryland, USA). The morphometric parameters evaluated included the length and width of the sperm head, as well as the length of the acrosome and nucleus (Gontarz et al., 2016).

### Statistical analysis

The results were expressed as mean and standard error of the mean. The normality of the residuals was assessed using the Shapiro-Wilk test, and homoscedasticity was evaluated using Bartlett's test. To compare the sperm parameters between groups, the Mann-Whitney test was used for variables that did not follow a normal distribution (hypoosmotic and broken tail), while Student's t-test was applied to those with a normal distribution (motility, viability sperm, normal spermatozoa, and coiled tail).

The use of different tests was justified by the fact that, during the verification of the assumptions of normality and homoscedasticity, some variables showed a normal distribution while others did not. The variables analyzed by the Mann-Whitney test (hypoosmotic and broken tail) were classified as non-parametric, as they did not present a normal distribution, thus requiring the use of this test. On the other hand, the variables analyzed by Student's t-test were considered parametric, since they exhibited a normal distribution and homoscedasticity, allowing the application of parametric tests. All analyses were performed using GraphPad Prism®, version 9.3 for Windows (GraphPad Software Inc., San Diego, CA, USA), adopting a significance level of 5%.

## Results

### Influence of phenotypes on sperm parameters

**Figure 1:**
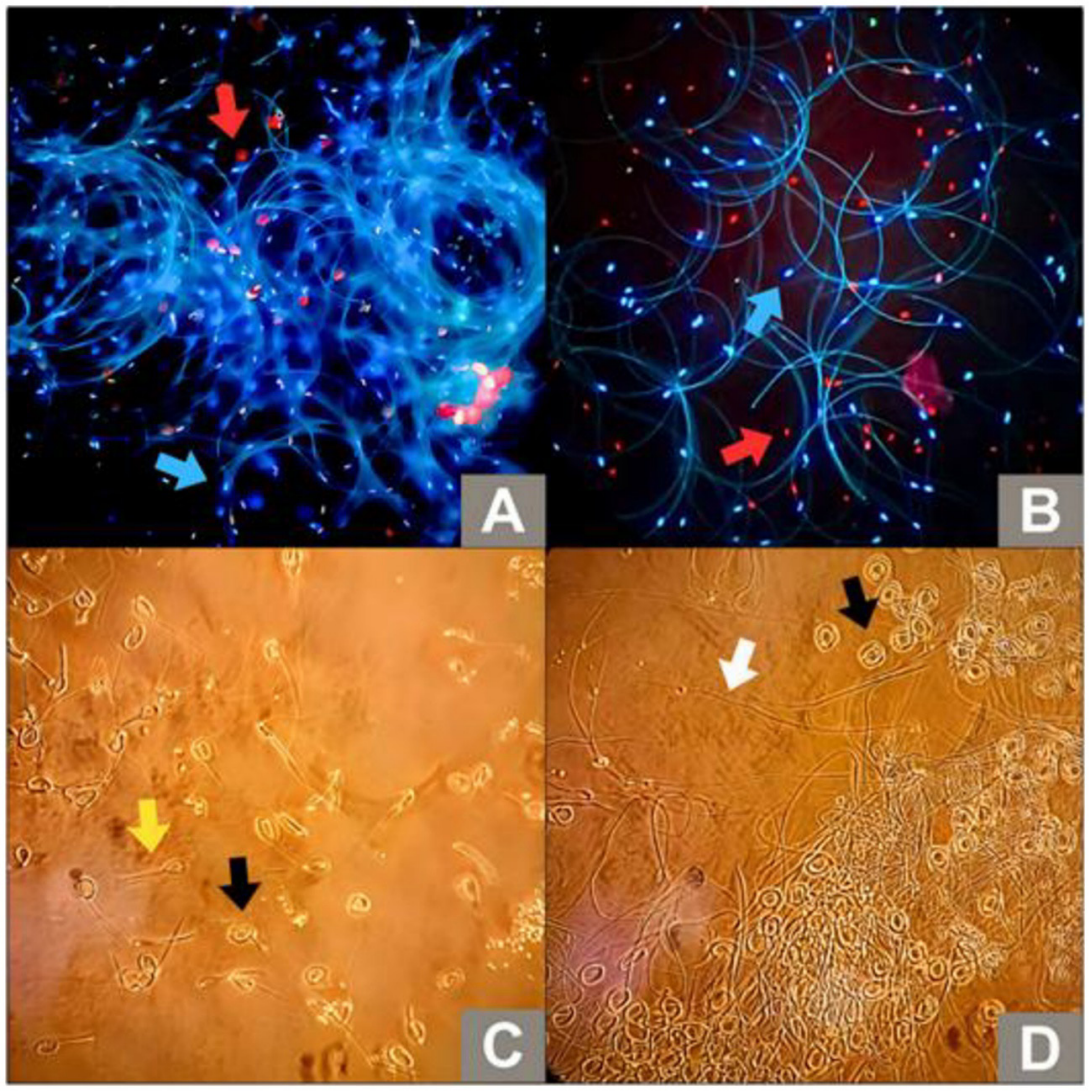
Sperm analyses of drones. (A) Sperm viability of Italian drone (*Apis mellifera ligustica*). (B) Sperm viability of Africanized drone (*Apis mellifera L.*); assessed by fluorescent probes: viable (blue arrow; Hoechst 33342) and nonviable sperm (red arrow; propidium iodide). (C) Functional integrity of the plasma membrane (hyposmotic test, HOST) of Italian drone (*Apis mellifera ligustica*); functionally intact membrane with slightly coiled tail (yellow arrow), functionally intact membrane with fully coiled tail (black arrow). (D) Functional integrity of the plasma membrane (HOST) of Africanized drone (*Apis mellifera L.*); compromised plasma membrane (white arrow), functionally intact membrane with fully coiled tail (black arrow).

Both phenotypes presented sperm motility around 90%, with sperm viability around 80% (Table 1). Both phenotypes exhibited sperm with circular movement patterns. In Italian drones, the functional integrity of the plasma membrane was 93.44 ± 1.7% while for Africanized drones it was 91.60 ± 1.5%, but no statistical difference was found between the phenotypes (P > 0.05), which were considered as presenting excellent sperm quality. Figure 1 illustrates the aspects related to the viability and membrane functionality of drone sperm from Africanized and Italian honeybees.

**Table 1:** Mean (mean ± SEM), for sperm motility, sperm viability and membrane functionality of Italian (*Apis mellifera ligustica*) and Africanized (*Apis mellifera L.*) honeybee drones.

**Semen Variables** ^*^	**Italian (n = 09)**		**Africanized (n = 16)**	
	**Mean** ± **(SEM)**	**Range**	**Mean** ± **(SEM)**	**Range**
**Motility (%)**	90.00 ± 0.0	90	90.00 ± 0.0	90
**Sperm Viability (%)**	82.44 ± 2.5	71-99	81.14 ± 2.4	65-94
**Membrane functionality (%)**	93.44 ± 1.8	84-98	91.60 ± 1.5	80-97

* There was no significant difference between phenotypes groups (P < 0.05).

### Influence of phenotypes on sperm morphology

**Figure 2:**
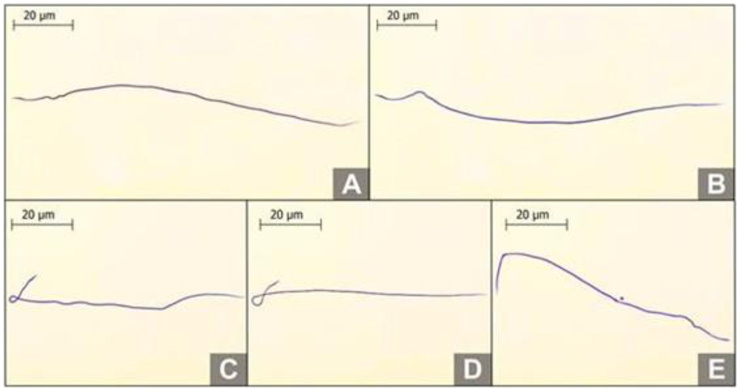
Morphological analyses of drone sperm stained with Rose Bengal. (A) Normal Italian sperm (*Apis mellifera ligustica*); (B) Normal Africanized sperm (*Apis mellifera L.*); (C) Normal sperm with a curled tail from an Italian drone (*Apis mellifera ligustica*); (D) Normal sperm with a curled tail from an Africanized drone (*Apis mellifera L.*); (E) Sperm with a broken tail from an Africanized drone (*Apis mellifera L.*).

Regarding sperm morphology (Figure 2), the percentage of normal sperm in Italian and Africanized drones was 10.89 ± 1.66% and 12.06 ± 1.01%, respectively, with no statistical difference, as shown in Table 2. Coiled tail was the most prevalent alteration observed for both phenotypes.

**Table 2:** Mean (mean ± SEM), for sperm morphology of Italian (*Apis mellifera ligustica*) and Africanized (*Apis mellifera L.*) honeybee drones.

**Semen Variables** ^*^	**Italian (n = 09)**		**Africanized (n = 16)**	
	**Mean** ± **(SEM)**	**Range**	**Mean** ± **(SEM)**	**Range**
**Normal morphology**				
Straight tail (%)	10.89 ± 1.7	4-20	12.06 ± 1.0	7-21
Curled tail (%)	88.56 ± 1.7	80-96	87.63 ± 1.1	78-93
**Altered morphology**				
Broken tail (%)	1.25 ± 0.3	1-2	1.00 ± 0.0	1

* There was no significant difference between phenotype groups (P < 0.05).

### Influence of phenotypes on sperm morphometry

There was a significant difference observed in the morphometry of the drones (Table 3). Morphometric measurements revealed that sperm from Italian drones had significantly (P < 0.05) larger heads (10.04 ± 0.03 µm) compared to Africanized drones (9.33 ± 0.04 µm), although the flagella and total length did not show significant differences, at approximately 260 µm in length in both groups.

**Table 3:** Mean (mean ± SEM), for sperm morphometry of Italian (*Apis mellifera ligustica*) and Africanized (*Apis mellifera L.*) honeybee drones.

**Semen Variables**	**Italian (n = 09)**		**Africanized (n = 16)**	
	**Mean** ± **(SEM)**	**Range**	**Mean** ± **(SEM)**	**Range**
**Morphometry**				
Head length (µm)	10.04 ± 0.03^a^	8.47-11.59	9.33 ± 0.04^b^	7.98-11.79
Tail length (µm)	249.7 ± 0.64^a^	221.3-298.3	250.2 ± 0.70^a^	222.0-301.5
Total length (µm)	259.7 ± 0.65^a^	231.4-308.3	259.9 ± 0.73^a^	230.3-311.2

a,b Different superscript lowercase letters in the same row (P < 0.05) represent statistical differences.

### Influence of phenotypes in scanning electron microscopy (SEM)

**Figure 3:**
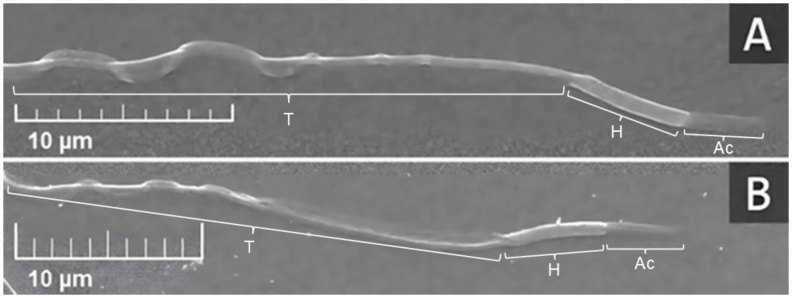
Ultrastructure of drone bee spermatozoa. (A) Italian (*Apis mellifera ligustica*); (B) Africanized (*Apis mellifera L.*). H: Head, Ac: Acrosome, T: Tail.

The ultrastructural evaluation of drone sperm (Figure 3) allowed detailed specification of the head, which is relatively small, thin, and narrow, measuring approximately 9 μm long and 0.86 μm wide in Italian drones and 8 μm long and 0.64 μm wide in Africanized drones. A conical acrosomal vesicle with a pointed, circular apex was observed, measuring approximately 3.37 μm long in Italian drones and 3.31 μm in Africanized drones, with a slight demarcation between the post-acrosomal region, which is delimited by the well-defined surface of the centrally convex, dense, elongated, and laterally flattened nucleus. This nucleus contains compact chromatin, measuring approximately 5.83 μm in Italian drones and 4.88 μm in Africanized drones, occupying most of the sperm head, giving the head a bean-pod appearance (Peng et al., 1992). In the presented head-flagellum, the main mitochondrial derivative exhibits asymmetry in length and diameter, with no differences identified between the groups. These details could not be clearly visualized by the conventional Rose Bengal staining method.

## Discussion

Male reproductive potential has a substantial effect on the population dynamics of a given species (Rankin and Kokko, 2007). Therefore, it is worth considering investigating different aspects of male fertility, including in bees. This study presents an unprecedented comparison of the sperm parameters of Italian and Africanized bee drones present in the same environment, the Caatinga biome. It was demonstrated that, in general, both phenotypes of drones present similar sperm characteristics, differing only in the morphometry of the sperm head. Furthermore, it is worth noting that both bees, when collected during the rainy season in the same semiarid environment, present excellent sperm quality.

Drone sperm motility stands out as one of the most relevant parameters, as it is directly related to the sperm's ability to migrate to the spermatheca and the success of fertilization (Yániz et al., 2020). Unlike what is observed in other species, drone sperm motility follows a predominantly circular movement pattern, with a helical shape (Tofilski et al., 2018; Murray et al., 2023). In the present experiment, both phenotypes presented that circular movement pattern, which is considered an indicator of sperm quality (Yániz et al., 2020). Moreover, both species presented 90% motile sperm on average, when collected during the Caatinga rainy season. These results are equivalent to those presented by Morais et al. (2022), who found averages of 85.6% of motile sperm in Africanized honeybee drones during the rainy season.

Regarding sperm viability, there were no statistically significant differences between the bee drones’ phenotypes analyzed. These results are like those reported by Morais et al. (2022), during the same rainy season. In the comparative study conducted by Kahya and Gençer (2023), which evaluated the reproductive quality of drones from Caucasian (*Apis mellifera caucasica*) and Italian (*Apis mellifera ligustica*) honeybees, no significant differences were found in the viability of spermatozoa within the seminal vesicles between the phenotypes. Sperm viability, in turn, is closely related to queen fertility and is widely used as an indicator of semen quality (Zhao et al., 2021). It is a fundamental parameter for drone fertility, since only sperm with intact plasma membranes can maintain cellular metabolism, reach the queen's spermatheca and effectively participate in the fertilization process (Yániz et al., 2020).

Motility and integrity of the sperm membrane (viability) are essential parameters for evaluating semen quality, both of which are directly related to the fertilizing potential of sperm. Maintaining an intact plasma membrane is essential to ensure the stability of metabolic functions, so that damage to this structure can compromise fertilization capacity (Yániz et al., 2013). In this context, joint analysis of motility and viability is essential, since sperm with damaged plasma membranes, although motile, may not be functionally competent for fertilization. Likewise, ejaculates with low sperm motility can compromise the animal's ability to reproduce (Aguiar et al., 2024). Membrane integrity is not only important for sperm metabolism, but a correct change in membrane properties is necessary for a successful union of male and female gametes. Thus, the integrity and functional activity of the sperm membrane are of fundamental importance in the fertilization process (Bratu et al., 2022).

Therefore, it is necessary that the sperm membrane structure is not only viable, but also functional. In this context, the use of a 0 mOsm/L solution proved to be effective in identifying the functional integrity of the plasma membrane, based on the degree of tail coiling (Nur et al., 2012). The results indicated good functional integrity of the plasma membrane in a 0 mOsm/L solution for both Italian and Africanized drones, with no statistically significant differences between the phenotypes. On the other hand, one point to be questioned is whether, although both phenotypes are functional and respond osmotically well to the 0 mOsm/L solution, there may be a difference in the composition of the sperm membrane, favoring a better adaptation to the female's physiological systems. Furthermore, in the solution present in the female's spermatheca, will the sperm respond in the same way or differently? These questions represent gaps that should be investigated in future studies.

Analysis of sperm morphology is an important indicator of sperm quality and fertilization success (Abu et al., 2012). Regarding the analysis of morphology, the drones of the Italian bee phenotype (*Apis mellifera ligustica*) and the Africanized bee (*Apis mellifera*) revealed typical species characteristics, with long, filamentous cells and tapered ends (Yániz et al., 2020). This morphology is common among bees of the *Apis* genus and has been associated with evolutionary adaptation for prolonged storage in the queen's spermatheca throughout her fertile life, as well as with sexual selection. Long spermatozoa have a greater capacity for energy production and higher efficiency in movement within the storage organ. Furthermore, sperm with greater longevity are more likely to fertilize eggs, making morphology and length highly selected traits (Slater, 2022).

For both Italian and Africanized drones, the occurrence of curled tails was the most predominant characteristic of morphological normality. The observation of coiled tails in sperm is related to the helical movement pattern exhibited by bee sperm (Tofilski et al., 2018; Murray et al., 2023). Therefore, this characteristic can be considered a consequence of the swimming pattern of these gametes. Furthermore morphological alterations may contribute to reduced fertilization rates (Larson-Cook et al., 2003).

The morphometric results demonstrated a significant difference in the size of the sperm head of Italian drones, which was larger compared to that of Africanized drones. This difference may be related to chromatin organization within the sperm nucleus, which directly influences head volume (Rathke et al., 2014). After meiosis and during spermiogenesis, round spermatids differentiate into mature spermatozoa. In this process, chromatin condensation occurs, during which nuclear volume is drastically reduced. Initially, proteins such as histones are added to the chromatin, but throughout sperm maturation, these are gradually replaced by small nuclear proteins called protamines. Chromatin condensation contributes to the formation of a compact, hydrodynamic nuclear structure, protects the genome from physical and chemical damage, and may be involved in epigenetic regulation (Rathke et al., 2014).

Considering that most of the sperm head is composed of highly compacted chromatin, the nuclear shape may be directly associated with the chromatin state of the spermatozoon (Saravia et al., 2007). Chromatin condensation is a fundamental process of DNA compaction and structural reorganization during spermatogenesis, and it is essential for the integrity and accurate transmission of genetic material (Vagnarelli, 2013). Therefore, it is possible that the spermatozoa of Italian drones have less condensed chromatin or a nuclear arrangement with a lower amount of DNA-stabilizing proteins, such as protamines, which would result in a larger sperm head. On the other hand, in Africanized drones, more compacted chromatin or a higher presence of nuclear proteins may contribute to a smaller sperm head, while still maintaining reproductive functionality. Thus, the morphometric differences observed in this study may reflect adaptations in genetic material packaging between the phenotypes analyzed, with possible implications for genomic stability, sperm longevity, and the queen’s reproductive success. Further specific studies on this topic are needed.

Although this difference in head size was observed, the length of the flagella and the total length of the sperm did not show significant variations between the groups. Overall, the mean morphometric values observed in this study are consistent with those described for sperm of European Buckfast bees found during the rainy season in central Romania (Bratu et al., 2022; Lino-Neto et al., 2000). It is worth mentioning that the experiment was extended in July 2024, at the end of the rainy season. However, the flagella and total length did not show significant differences between the groups.

Unlike in vertebrates, insect spermatozoa do not exhibit a readily distinguishable midpiece. They are filamentous, with an elongated head and an extremely long tail, composed of a flagellum derived from the axial filament and two mitochondrial structures surrounding it: a larger mitochondrial derivative, located closer to the nucleus, and a smaller one, which complements these structures along with the accessory bodies (Peng et al., 1993; Lino-Neto et al., 2000). This configuration can be observed through scanning electron microscopy analyses of drone bees from Italian and Africanized strains, which allowed for the specific identification of the conical acrosomal vesicle and the well-defined surface of the nucleus, although the surrounding component was not visualized. In the analyzed head-flagellum region, the main mitochondrial derivative was observed; however, no differences were identified between the groups.

This study, which evaluates the sperm parameters of drones by comparing different phenotypes, contributes to a more precise understanding of the morphological and functional characteristics of spermatozoa. It serves as a basis for the development of cryopreservation and artificial insemination protocols, which, in the long term, ensure the storage of genetic material and allow the preservation of the characteristics of the species.

## Conclusions

In conclusion, there is great similarity related to the sperm parameters of Italian (*Apis mellifera ligustica*) and Africanized (*Apis mellifera L.*) drones raised in Caatinga biome. This information contributes to understand the reproductive physiology of the phenotypes and emphasizes the possibility of applying similar reproductive managements for them, allowing the development of strategies for these bee phenotypes conservation.

## Data Availability

Research data is only available upon request.
